# Dogs’ Body Language Relevant to Learning Achievement

**DOI:** 10.3390/ani4010045

**Published:** 2014-02-27

**Authors:** Masashi Hasegawa, Nobuyo Ohtani, Mitsuaki Ohta

**Affiliations:** Azabu University School of Veterinary Medicine, 1-17-71, Fuchinobe, Chuo-ku, Sagamihara, Kanagawa 252-5201, Japan; E-Mail: mohta@azabu-u.ac.jp

**Keywords:** body language, *Canis familiaris*, learning, operant conditioning, training

## Abstract

**Simple Summary:**

For humans and dogs to live together amiably, dog training is required, and a lack of obedience training is significantly related to the prevalence of certain behavioral problems. To train efficiently, it is important that the trainer/owner ascertains the learning level of the dog. Understanding the dog’s body language helps humans understand the animal’s emotions. This study evaluated the posture of certain dog body parts during operant conditioning. Our findings suggest that certain postures were related to the dog’s learning level during operant conditioning. Being aware of these postures could be helpful to understand canine emotion during learning.

**Abstract:**

The facial expressions and body postures of dogs can give helpful information about their moods and emotional states. People can more effectively obedience train their dogs if we can identify the mannerisms associated with learning in dogs. The aim of this study was to clarify the dog’s body language during operant conditioning to predict achievement in the test that followed by measuring the duration of behaviors. Forty-six untrained dogs (17 males and 26 females) of various breeds were used. Each session consisted of 5 minutes of training with a treat reward followed by 3 minutes of rest and finally an operant conditioning test that consisted of 20 “hand motion” cues. The operant tests were conducted a total of nine times over three consecutive days, and the success numbers were counted. The duration of the dog’s behavior, focusing on the dog’s eyes, mouth, ears, tail and tail-wagging, was recorded during the operant conditioning sessions before the test. Particular behaviors, including wide-eyes, closed mouth, erect ears, and forward and high tail carriage, without wagging or with short and quick wagging, related to high achievement results. It is concluded that dogs' body language during operant conditioning was related to their success rate.

## 1. Introduction

Dogs, which were probably the first domesticated animals, have shared a common environment with humans for over ten thousand years [1]. Hare’s 2002 study did not support the predictions of either the canid generalization hypothesis, which states that dogs have inherited their skills from wolves, or the human exposure hypothesis, which states that dogs are skillful because they experience intense exposure to humans through their lives [2]. Instead, the study provided support for the domestication hypothesis: that dogs acquired their social-communicative skills with humans during the process of domestication [3,4,5]. Dogs and humans are capable of a unique interspecies communication, and understanding, and share basic emotions [6]. Over the last decade, many studies have shown that dogs respond to human actions, which might be an effect of domestication [7,8,9,10,11]. These reports have demonstrated that dogs understand and responded to human gestures and cues, such as pointing, head turning, gazing and nodding, to locate hidden food items.

Dogs have three main methods of communicating with others: auditory, visual and olfactory [12]. The visual communication methods, including postures and facial expressions, are known to be descended from those of wolves, their ancestor [13]. However, the selection by humans for particular physical features has hampered the dog’s abilities to use certain structures for visual communication [14,15]. For example, drooping ears and/or docked tails may be less able to signal the dog’s status than more wolf-like conformations [12]. There are more than 400 canine breeds differing in external morphology and behavior [16,17,18]. Furthermore, there is a great deal of variability in the social behavior towards humans and the communicative behavior due to the breeds [19,20]. For example, it is much easier to teach a Labrador than a Great Pyrenees to retrieve [21]. However, no dog is immune to the principles of learning.

In Japan, nearly 24% of households have pet dogs, resulting in a total of over 10 million dogs, and this is almost the same as the number of children under 14 years of age [22,23]. The more humans that have close ties to dogs, the more dog behavioral problems become a serious issue. Many of the problem behaviors arise from the types of relationships people have with their dogs [24,25]. Obedience training is usually recommended for dogs displaying overexcitement [19,26]. Additionally, the timing of meals and sleeping arrangements, as well as the owners’ prior experiences with dogs and their reasons for acquiring a dog, are all significantly related to the prevalence of certain behavioral problems [27]. Thus, we need to increase the awareness of the importance of appropriate dog obedience training in Japan. 

The obedience training of domestic dogs, and indeed other animals, to perform behaviors on command is well established using operant conditioning and Pavlovian conditioning techniques [28,29]. Many trainers utilize the method of luring a dog by hand into a position using food or toys, marking the behavior with a clicker or voice cue, and offering a reward for learning a new obedience behavior, such as sitting [30]. The percentage of dog owners using the reward method for sit training was identified as 75% through questionnaires [31].

For effective training, it is necessary that the dog is highly motivated and concentrating to enable it to learn, and that the owner has the appropriate training skills. Dog owners may not have the time or ability to acquire the skills of a dog trainer. However, the owners can perform more effective dog training if they can read, by some kind of method, the dog’s emotional and motivational states as well as their attention span for learning. 

In humans, the importance of non-verbal signals during verbal exchanges is widely recognized [32]. Although emotion recognition includes expressions and gestures, facial expressions are uniquely relevant, and the correct interpretations of facial expressions are needed for appropriate emotional reactions and appropriate behavior in social situations [33]. A number of studies indicated that perceivers were more influenced by visual cues, particularly the facial expressions of humans, than by a vocal component [34,35,36]. Thus, it is natural that we humans try to understand dog emotions through their visual cues. On the other hand, although some studies have shown the meaning of various facial expressions and body postures of canids [13,37,38], there is little information on the relationship between body language and learning level.

Thus, the aim of this study was to elucidate the common body language in a variety of breeds relevant to learning achievement by operant conditioning. Our results may establish a relationship between body language and learning achievement in dogs. 

## 2. Experimental Section

### 2.1. Ethics

All of the procedures were approved by the Animal Experiments Ethics Committee of Azabu University in accordance with the World Medical Association Declaration of Helsinki.

### 2.2. Subjects

Forty-six dogs (17 males and 26 females) of various breeds, kept at the World Ranch in Osaka, Japan, participated in this study (Table 1). All of the dogs were sexually intact and between 12 to 79 months of age (average 35.9 months, not including three dogs whose ages were unknown). They were housed in individual 120 cm × 200 cm metal cages from 17:00 to 9:00 away from human contact and provided with commercial dog food (Adult Maintenance, Nutro Products Inc., Victorville, CA, USA), according to the industry recommendations. Food was given twice a day, at 8:00 in the morning and at 17:00 in the evening. Furthermore, all of the dogs were released outside at the same facility from 9:00 to 17:00 in an exercise yard, which was surrounded by a 200 cm × 300 cm metal fence for at least one year. They had been socialized through interactions with people of various ages that visited the ranch. 

Additionally, the kennel staff confirmed that the dogs had not been trained nor did they respond to a verbal sit command or the associated hand motion. As a result, dogs that did not respond to either stimuli became experimental subjects.

**Table 1:** Characteristics of dogs *(Canis familiaris*) used in the present study.

Breed	Sex	Age (months)		Breed	Sex	Age (months)
*Australian Terrier*	♂	62		*Irish Setter*	♂	49
*Australian Cattle Dog*	♂	54		*Irish Wolfhound*	♂	66
*Australian Kelpie*	♂	67		*Irish Wolfhound*	♀	79
*Basset Hound*	♀	49		*Japanese Spitz*	♀	12
*Beagle*	♂	12		*Miniature Pinscher*	♀	12
*Beagle*	♀	12		*Miniature Schnauzer*	♂	27
*Border Collie*	♀	12		*Newfoundland*	♀	30
*Boxer*	♀	25		*Newfoundland*	♂	77
*Boxer*	♂	36		*Newfoundland*	♀	30
*Bulldog*	♀	48		*Norfolk Terrier*	♂	12
*Chihuahua*	♀	66		*Papillon*	♀	13
*Chihuahua*	♀	12		*Papillon*	♀	12
*Clumber Spaniel*	♀	12		*Pug*	♀	54
*Dandie Dinmont Terrier*	♂	UN		*Pug*	♀	24
*English Cocker Spaniel*	♀	17		*Saint Bernard*	♀	55
*English Cocker Spaniel*	♂	45		*Schipperke*	♂	UN
*English Setter*	♀	41		*Shar pei*	♂	25
*English Springer Spaniel*	♀	40		*Shetland Sheepdog*	♀	49
*French Bulldog*	♀	17		*Vizsla*	♂	39
*French Bulldog*	♂	24		*West Highland White Terrier*	♀	12
*Greyhound*	♀	73		*Yorkshire Terrier*	♂	36
*Ibizan Hound*	♀	UN		UN: Unknown		

### 2.3. Training Procedure

The training and experiments were conducted in the dog’s familiar exercise yard by a handler (male, 28 years old, accustomed to working with dogs). The exercise yard was 150 cm × 200 cm and distracting smells were eliminated by cleaning as much as possible. The experiments were not affected by visual and auditory stimuli. The dogs and the handler had never met before the first training session. The treat used was a multi-balanced dog food (330 kcal/100 g, Nisshin Co.), stored in a bag kept at a constant humidity and temperature. The handler ascertained that all the dogs followed the hand with the treat before the training sessions began. 

In the 5 min training of the dogs, the handler, holding one piece of food between his thumb and forefinger, lead the dog to sniff his hand in situations where the dog was free in the exercise yard. With the dog’s nose on the handler’s fingers, the handler slowly moved his hand up and over the dog’s head. The operant conditioning was repeated as much as possible during the 5 min training sessions. The handler conditioned the dog to follow the hand motion. Thus, a hand motion from the dog’s nose to overhead became a discriminative stimulus and induced sitting behavior as an operant response. However, to avoid associating any irrelevant motion with the sitting behavior, the handler did not start the hand motion if the dog was not interested in his hand. Thus, the hand motion was only performed when the dog was likely to follow the hand and experience success.

During each operant conditioning test, the dog was given the same discriminative stimuli (the hand motion) 20 times, and the number of successes was counted. Since the food might be the visual prompt for the dogs to sit, and prompt must be gradually be eliminated so that the animal learns to perform the behavior without the prompting [39], the handler did not have the food in his hands when he tested the dogs. In the test, we ascertained that just the hand motion was the discriminative stimulus to sit.

Continuous reinforcement was performed in all of the 5 min training sessions *and the test*, and a partial or intermittent reinforcement schedule was never used to avoid extinction. Thus, we carefully performed the operant conditioning sessions and the test to avoid extinction by rewarding every instance of sitting in response to the hand cue during training and test conditions. 

One session consisted of 5 min of training with the treat reward and an operant conditioning test that consisted of 20 “hand motion” cues followed after 3 min of rest. Dogs had three sessions per a day, with 3 minutes of rest in between each session. This training schedule of a day (Table 2) was repeated for three consecutive days (leading to nine sessions per dog) in the afternoon prior to the evening feeding time. 

**Table 2:** The daily training schedule.

	Session 1		
Time	5 min	3 min	hand motion 20 times
Detail	Training with treat	Resting	Operant conditioning test

### 2.4. Behavioral Evaluation

**Figure 1:**
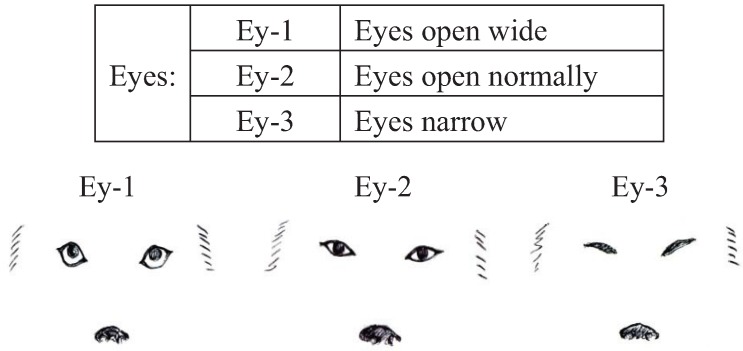
Categories of eyes (Ey) in dogs during their training.

**Figure 2:**
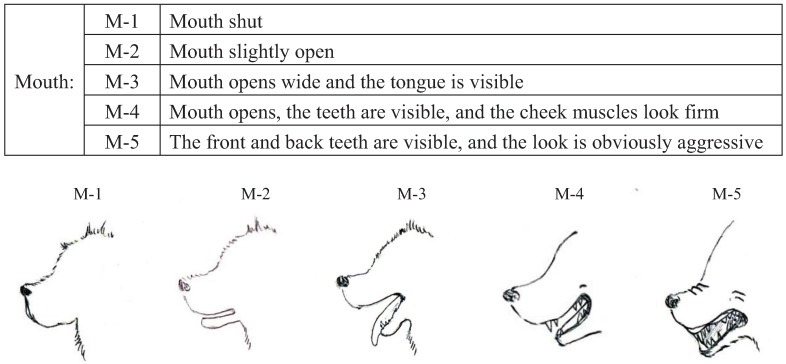
Categories of mouth (M) expressions in dogs during their training.

**Figure 3:**
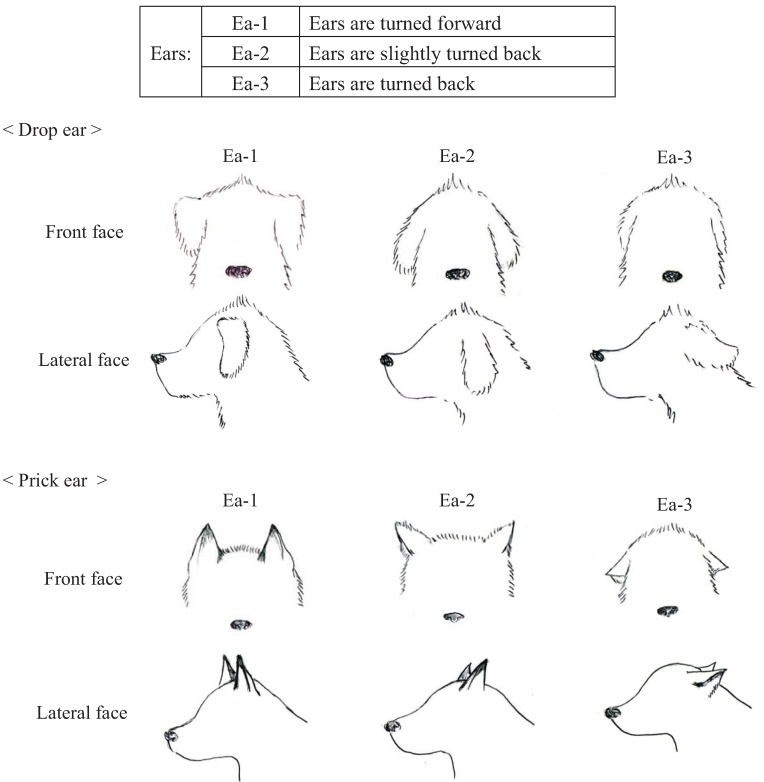
Categories of ear (Ea) appearances in dogs during their training.

**Figure 4:**
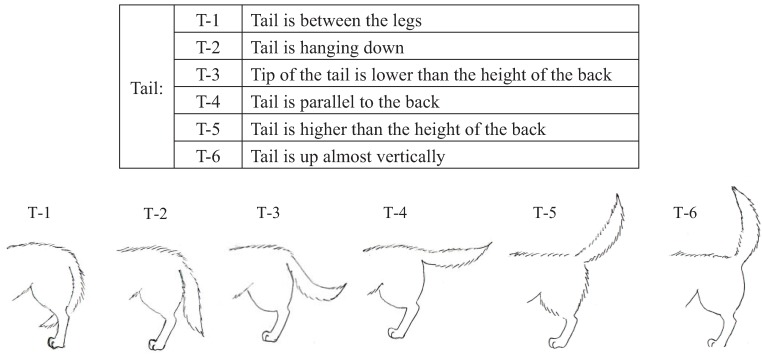
Categories of tail (T) body language in dogs during their training.

We conducted pre-experiments on 10 other dogs before this main experiment. This pre-experiment was carried out for three days with training schedules that were exactly the same as the main experiment. The sessions were recorded using a digital video camera (DCR-HC90, Sony, Japan). We categorized the body language of the dogs using the videos and many references [12,40,41,42,43]. Body languages were frequently observed in the videos and were given simple classifications that a dog owner could recognize. Eyes (Ey) and Ears (Ea) were divided into three categories based on their appearance. In addition, the types of ears were broadly classified into drop ear and prick ear. Mouth (M) appearance was divided into five categories. The Tail (T) category recorded tail height from the lowest to highest position. Tail-Wagging (TW) was also classified based on the visual records. The behavioral categories are shown in Figure 1, Figure 2, Figure 3, Figure 4 and Table 3. 

**Table 3:** Categories of dog tail-wagging (TW) during operant conditioning training.

Tail-Wagging:	TW-1	Tail is not wagging
	TW-2	Only the tip of the tail is wagging
	TW-3	Tail is wagging shortly and slowly
	TW-4	Tail is wagging shortly and quickly
	TW-5	Tail is wagging widely and slowly
	TW-6	Tail is wagging widely and quickly
	TW-7	Tail is wagging with wiggling at the hip and waist
	TW-8	Tail is spinning around

In the main experiment, three experimenters recorded observations independently for each behavioral category, Eyes (Ey), Mouth (M), Ears (Ea), Tail (T) and Tail-Wagging (TW), during the nine training sessions excluding the operant conditioning tests. The three observers had been trained in the methods of behavioral evaluations of dogs. The behavioral assessments were performed by analyzing slow motion replays of the 5 min of taped training. These evaluations lasted approximately 25 min. However, we have excluded from the records of the tail movements that could not be evaluated, such as those of the Pugs.

### 2.5. Statistical Analysis

The number of the operant conditioning events during the 5 min training sessions, and of the successful operant responses after the conditioning, were counted. It was necessary to identify any correlation between the conditioning number in the training and the success number in the test. Since the age of subject dogs ranged from 1 to 6.5 years old in this experiment, there could be age-dependent differences in the learning acquisition rate. Therefore, the correlation between the conditioning number and success number, and the test results and the dogs’ ages, were analyzed using Spearman's rank correlation coefficient.

Additionally, the three experimenters recorded the duration of the body language categories, thus the inter-experimenter reliability was established using Cronbach’s alpha analysis. 

This study was done to clarify the body language of dogs that performed well during operant conditioning. Therefore, a stepwise multiple regression analysis was performed to determine whether the duration of each of the behavioral categories was a good predictor for high achievement during the operant conditioning test. The high achievement dogs showed more than the median split number of successful operant responses. And the stepwise multiple regression was used to avoid problems of multicollinearity. In this analysis, the significant variables were selected using backwards elimination, and the categories of body language were independent variables and learning achievement in the operant conditioning test was the dependent variable. The statistical analysis was performed using the computing software R (http://www.r-project.org). 

## 3. Results

There was a significant positive correlation between the number of conditioning events and the success number of the test (rs = 0.7, *p* < 0.05). There were no statistical correlation between the age of dogs and results of operant conditioning test (rs = −0.31, *p* = −0.35). Moreover, inter-experimenter reliability was confirmed using Cronbach’s alpha analysis (α = 0.83). 

The median split number of success responses in the test was 9. The stepwise multiple regression analysis is shown in Table 4. The results indicated that the significant traits were Ey open wide (standard partial regression coefficient = 0.14, *P* < 0.05), M shut (standard partial regression coefficient = 0.48, *P* < 0.001), Ea turned forward (standard partial regression coefficient = 0.19, *P* < 0.01), T is hanging down (standard partial regression coefficient = −0.16, *P* < 0.05), T is up almost vertically (standard partial regression coefficient = 0.18, *P* < 0.01), TW is not wagging (standard partial regression coefficient = 0.34, *P* < 0.001), and TW is wagging short and quick (standard partial regression coefficient = 0.33, *P* < 0.001).

**Table 4:** Statistical results on the duration of each behavioral category as good predictors of the achievement rate in the operant conditioning tests (Stepwise multiple regression analysis).

Variables		*β*	t
Eyes	Ey-1	0.14 *	2.01
Mouth	M-1	0.48 ***	7.15
Ears	Ea-1	0.19 **	2.58
Tail	T-2	-0.16 *	2.1
	T-6	0.18 **	2.44
Tail-Wagging	TW-1	0.34 ***	3.79
	TW-4	0.33 ***	4.79

* P < 0.05,** P < 0.01, *** P < 0.001.

## 4. Discussion

To clarify the body language of dogs with high learning performances, we recorded the duration of each behavioral category during nine training sessions over three consecutive days. This study revealed, by detailed behavioral evaluation and statistical analysis, that some of the dog’s body language correlated with high achievement in the operant conditioning tests. Wide eyes and upright ears have been observed in dogs that are expressing aggression or dominance, and in those that feel challenged or threatened [40]. In this study, the wide-open eyes were linked with high learning results, which suggests that some of the dogs were expressing dominance over humans. A dominant dog shows a self-assured gait, a large, confident body posture, raised head, raised ears, large eyes, curled lips, and carries the tails high with a slight wag [40]. However, the body language categories linked to a high learning performance were closed mouths, forward ears, and high tail carriage without wagging. This is similar to the body language of dominant dogs, but was different in the categories of mouths (lips) and tail-wagging, which suggests that the dogs that recorded high results in the test were not dominant over the experimenter. 

Humans learn many things by eyesight, and eye contact is essential for developing social skills [44]; however, in dogs, staring eye-to-eye means opposition [40]. Submissive dogs avoid direct eye contact with the dominant dog, and it has been suggested that the relative status of the dogs is determined by this visual communication [14]. In this study, wide eyes were observed mostly when the dogs looked up at the handler’s face. The tendency of dogs to have periods of face/eye contact with people longer than socialized wolves in the food task was observed [45]. Thus, dogs naturally do not watch other dog’s eyes; however, it is thought that an effect of domestication is that dogs will watch a person’s eyes. Furthermore, while face/eye contact indicated the superior and inferior relationship between dogs [40], looking up at a human face and making eye contact can be categorized as body language focusing or expectation on a human.

Although the dog’s body language that was linked to a high learning performance included the category of closed mouth in this study, as the training period progressed, in general, dogs start panting to maintain their body temperature. Additionally, even if the dog was socialized, the experimenter was a stranger to the dogs in the exercise yard. Since excessive stress may induce dogs to perform thermoregulatory behavior like panting [46], it may be that dog’s panting was started by stress of the stranger. Therefore, it may be difficult to discriminate how much dogs are focusing on people just by watching their mouths.

The duration of the ears pointing forward was relevant to a high learning performance. The normal body language of a dog when greeting a human or another dog includes the movement of its ears up and down [40]. However, a forward ear position is associated with a state of heightened attention [47], motivation, confidence, and/or aggression; whereas, a backward ear position is often associated with submission and/or fear [40]. These results suggest that while the dogs are focused on the handler, their ears point towards them.

In the category of the tail height, the duration of the tail being held straight up was linked with the dog’s high response during the test. In the category of tail wagging, it was revealed that a non-wagging tail or a tail wagging shortly and quickly could predict a high learning performance during the training. It may be suggested that the position and wagging of the tail represent the motivation status of the dog. However, the shapes and sizes of the tails differ in all breeds and there are breeds that cannot bring their tails up straight [40]. Thus, such an index can only be used for dog breeds that are able to bring their tails up straight and may not apply to some breeds that have specific types of tails. However, the combination of the position and motion of a domestic dog’s tail still provides information regarding the dog’s state, including friendly, playful, fearful, submissive, dominant and aggressive [12,42,48,49,50]. Therefore, a study that focuses on only the tail position and wagging should be conducted in each breed. Quaranta *et al*. studied the asymmetric tail-wagging responses of dogs to different emotive stimuli [51]. In their study, the brain activity occurring when the dog’s tail was wagged to the left or right was discussed. Although we did not analyze the direction of tail wagging in our study, further studies may be able to explain the brain activity during training. 

One question that remains is what it was about the low achieving dogs that caused them to learn more slowly. All of the dogs were given treats in the 5 minutes of training and the operant conditioning test using continuous reinforcement. Thus, the training and test conditions were not to extinction. However, in the test, the handler gave the same hand motion 20 times as a discriminative stimuli to the dogs, regardless of their learning, when the dogs looked at the handler’s hand. We believe that these dogs are displaying learned irrelevance. Learned irrelevance, learning to ignore things that are of no importance [39], is a particularly efficient way of learning. Therefore, it might take more time for the low achievement dogs in the test to learn that the hand motion was a discriminative stimulus to sit cue. In other words, the low achievement dogs might have needed to increase the frequency of training.

Additionally, there might be other causes of the slow learning in dogs that recorded low achievement numbers, such as breed differences/behavior, and changes in motivation due to satiation (treats). Hart and Hart examined the breed behavior profiles of 56 breeds of dogs using 13 behavioral traits, and elucidated that the obedience training performance was affected by the dog’s breed [19]. We had 33 breeds in our experiment, and it is possible that there might be a difference in learning performance between these breeds. However, there were too few of each dog breed to perform a statistical analysis that would ascertain the ability for training between dog breeds. Additionally, we gave each dog one treat for each event during the operant conditioning. The treat size was standard and not based the size of the dog. Thus, we did not change the size of the treat to account for the dog’s feeling of satiation. The total number of operant conditioning events for the Newfoundland was 130, the Papillion was 194, and the Chihuahua was 141 in this experiment. It appears that the number of operant conditioning events was unaffected by the size of the treats. However, to conduct a more detailed experiment and be able to do a statistical analysis, it will be necessary to increase the number of dogs per breed to investigate the relationship between dog breed and learning performance.

Body language shows the dog’s status, such as dominant and submissive [40]. In this study, an unknown handler trained dogs that had not lived with human owners. Thus, they probably they had various emotional responses to the handler, and their body language may differ from the general home dog. However, using these body language parameters in our dog school for pet dogs, the learning results were improved. To evaluate the usefulness of these traits, it would be necessary to perform the same evaluation of dogs living with their owners.

The body language of dogs linked with high test results was very similar to the body language of a dominant dog. However, there were differences in the categories of mouths (lips) and tail-wagging. The dominant dog watches other dog closely [14], and the dogs that recorded high learning results were also looking up at the human face/eyes. Thus, it may be that the categories of eyes open wide and ears turned forward, without the categories of mouths (lips), tail and tail-wagging, mean attention to others. 

Thus, wide eyes and forward ear positions, which were the categorized body language in this study, determined the common body language in a variety of breeds relevant to learning achievement during the operant conditioning. This body language will become the key to understanding the level of motivation and attention in the operant conditioning of dogs. However, in the dog’s body language each emotion is expressed using the whole body. Thus, elucidation of the entire body language, not just those related to the eyes and ear position, is required, especially the dog’s tail position/wagging in each breed.

## 5. Conclusions

During dog training, the common dog body language among some breeds that predicted high learning achievement was wide-open eyes, ears erect and having a forward tail position. These traits could aid in the efficient training of dogs. Additionally, the mouth appearance was affected by temperature and stress, and the category of the tail needs to be examined in a breed-based manner.
